# Editorial: Cancer cell reprogramming: Impact on carcinogenesis and cancer progression

**DOI:** 10.3389/fonc.2023.1152402

**Published:** 2023-02-15

**Authors:** Yue Zhao, Simona Kranjc Brezar, Elvira V. Grigorieva, Ira-ida Skvortsova

**Affiliations:** ^1^ Department of General, Visceral, Tumor and Transplantation Surgery, University Hospital of Cologne, Cologne, Germany; ^2^ Department of Experimental Oncology, Institute of Oncology Ljubljana, Ljubljana, Slovenia; ^3^ Institute of Molecular Biology and Biophysics Federal Research Center of Fundamental and Translational Medicine (FRC FTM), Timakova, Novosibirsk, Russia; ^4^ Laboratory for Experimental and Translational Research on Radiation Oncology (EXTRO-Lab), Department of Therapeutic Radiology and Oncology, Innsbruck Medical University, Innsbruck, Austria

**Keywords:** cell reprogramming, cancer stem cell, cancer metabolism, cancer metastasis, carcinogenesis

The transformation of normal cells into cancerous cells is a multi-stage process which progresses from a pre-cancerous lesion to a malignant tumor. There were an estimated almost 10.0 million deaths from cancer worldwide in 2020 (1). Currently challenges in cancer managements major due to late diagnosis, metastasis, recurrence, tumor heterogeneity and therapy resistance (2), All these failures could be explained by the characteristics of tumor heterogeneity and the existence of cancer stem cells (CSCs) (3). The concept of cellular plasticity was first proposed by Gurdon et al. (4). Cancer cells are also showing genetically and epigenetically plasticity, so cancer cell reprogramming could cover a board of cellular and molecular alterations, having significant impact on carcinogenesis and cancer progression and will also provide promising therapeutic strategies to convert the tumor malignancy (5, 6). In this Research Topic: ‘*Cancer Cell Reprogramming: Impact on Carcinogenesis and Cancer Progression*’, we aim to collect and discuss the studies on this topic based on 5 review articles, 13 original articles and 7 bioinformatic centralized studies.

First of all, signaling pathways and key modulators in cancer reprogramming are still the hot topics in cancer research. Knowing biological targets or biomarkers enables us to develop an effective and specific treatment approach in melanoma, lung, gastric and hepatocellular cancer (7, 8). Zhang et al. demonstrated the NAP1L1 as a potentially significant oncogene that could be used as a prognostic factor in hepatocellular carcinoma (HCC). Indeed, the upregulation of NAP1L1 promotes disease progression and predicts poor prognosis of HCC in patients. Silencing of NAP1L1 expression significantly decreased cell proliferation and cell cycle transformation *in vitro* and *in vivo*. The reversion of these suppressive effects in NAP1L1 cells after transfecting HDGF or c-Jun, clearly indicates for the first time NAP1L1 as a potential oncogene that acts *via* HDGF/c-Jun signaling pathway in HCC.

Despite different treatment approaches, surgery, immunotherapy, radiation therapy, chemotherapy, or combination therapy, malignant melanoma is the most invasive and fatal skin carcinoma. Thus, the time of melanoma diagnosis and the use of specific biological markers are crucial. Wang et al. focused on a gene encoding a subtype of Latent transforming growth factor binding protein 4 (LTBP4), an extracellular matrix glycoprotein belonging to the LTBP/fibrillins superfamily that is involved in the development of skin melanoma. Down-regulation of LTBP4 expression in cells and melanoma tissues predicted poor prognosis of melanoma patients. Furthermore, the authors indicated the crucial role of the molecular signaling pathway LTBP4-TGFβ1-Hippo-YAP1 in the progression of skin melanoma *in vitro* and *in vivo* by switching it on/off. LTBP4 may function as a possible new biomarker for melanoma skin.

Nowadays treatment options for gastric cancer are still limited (9, 10). Resistance to chemotherapeutic drugs represents a great challenge in cancer treatment. To provide survival benefits to patients with oxaliplatin combined therapies, Li et al. studied the mechanisms of resistance to oxaliplatin in human organoids grown *in vitro* and subcutaneously *in vivo* and compared the data to patients’ follow-ups. This study greatly represents the development of personalized therapy. Overexpressed PARP1 was shown as an important gene involved in oxaliplatin resistance by inhibiting the base excision repair pathway. Additionally, for the first time, it was shown that oxaliplatin inhibited homologous recombination by CDK1 activity and made cancers with normal BRCA1 function sensitive to PARP inhibition. Importantly, they demonstrated that combining oxaliplatin with a PARP1 inhibitor can overcome the oxaliplatin resistance, suggesting a potential new treatment modality for patients with gastric cancer without BRCA1 mutations. This observation can be helpful in clinical practice to evaluate the efficacy of PARP1 inhibitors in combination with platinum compounds in gastric cancer patients.

The new aspect in the personalized medicine of gastric cancer is focused on targeting the tumor microenvironment which includes a variety of cellular and non-cellular components such as non-malignant host cells, immune, blood, and endothelial cells, fibroblasts, mesenchymal stromal cells, and extracellular matrix. Unfortunately, targeting drugs for the first-line treatment of gastric cancer are still unavailable. Liu et al. assume that the reason for this situation is due to the lack of the biomarkers and complexity of the tumor microenvironment in gastric cancer. The authors suggested to consider the Neuronal Regeneration Related Protein (NREP) as a key regulator of gastric cancer progression. It was shown that NREP is involved in EMT activation, CAF mibilization, actin cytoskeleton remodeling, and M2 macrophage infiltration resulting in tumor development and progression.

Non-small cell lung cancer (NSCLC) represents approximately 85% of subtypes of lung cancer, which is the leading cause of cancer death worldwide. In this issue, Guo et al. elucidated autophagy, an important physiological activity that controls cell survival and death, affecting cell homeostasis and clinical therapeutics. The authors give us a nice historical overview through the preclinical and clinical data in NSCLC demonstrating that autophagy modifies the tumor microenvironment, participates in metabolic reprogramming, eliminates ROS, promotes resistance to chemotherapeutic drugs and tumor evasion in antitumor immune responses. Additionally, a brief description of autophagy biomarkers NSCLC and already used agents in the inhibition of autophagy enlighten us on how to further develop new treatment options for NSCLC. In a translational study of lung adenocarcinoma, Hao et al. found that knock down of RFWD2, an E3 ubiquitin ligase, may reverse the oncogenic role of the tribble (TRIB) pseudokinase protein family member- TRIB2. TRIB2 was reported as an oncogene and promoted cancer cell proliferation and migration (11). In this study, they further demonstrated that TRIB2 regulated the lung cancer cell growth by modulating the proteasome-mediated degradation of proteins *via* interaction with RFWD2.

Recently, continuously growing number of studies on the role of the mitochondrial Ca^2+^ in the progression of malignant tumors leads to the increasing interest of basic researchers to investigate the molecular regulation of mitochondrial calcium homeostasis in cancer progression. It is currently known that mitochondrial Ca^2+^ uptake is markedly enhanced in a variety of malignancies, but the exact mechanisms orchestrating the levels of Ca^2+^ in mitochondria are not fully elucidated. Zhao et al. have shown that Ca^2+^ uptake can be upregulated by the mitochondrial calcium uniporter (MCU). Augmented Ca^2+^ concentration results in increased mitochondrial biogenesis accompanied by suppressing the phosphorylation of mitochondrial transcription factor A (TFAM). Authors have focused on the molecular mechanisms underlying the inhibition of TFAM phosphorylation by MCU-related mitochondrial Ca^2+^ uptake. It was found that PDE2/cAMP/PKA axis contributed tot he TFAM stability and colorectal cancer cell growth.

Several articles are devoted to the study of molecular mechanisms of the development of such aggressive urologic tumors as clear cell renal cell carcinoma (ccRCC) and the search for new biomarkers for proper adjuvant therapy selection (12, 13). Li et al. demonstrated the involvement of a poorly studied member of the methyltransferases family - METTL7B in ccRCC tumorigenesis. METTL7B knockdown inhibited the growth of ccRCC *in vitro* and *in vivo* by inhibiting the expression of genes involved in the regulation of cell cycle and invasion, for the first time identifying METTL7B as a biomarker of poor clinical outcome and potential therapeutic target in ccRCC patients.

In addition, Ren et al. paid special attention to the genes, which are downregulated in ccRCC, so not being cancer biomarker candidates, although the genes are also important for carcinogenesis. The authors demonstrated that transcriptional repressor zinc-finger protein 304 (ZNF304) is down-regulated in ccRCC tissue, and a lower level of this protein is associated with poor prognosis of patient survival. A molecular mechanism of ZNF304 involvement in RCC progression is realized through ZNF304/miR-183-5p/FOXO4 axis, suggesting key components of this pathway as potential therapeutic targets in ccRCC.

Metabolic reprogramming of cancer cells is one of the most important hallmarks of cancer (14). Yao et al. demonstrated the important role of signal-induced proliferation-associated 1 (SIPA1) in aerobic glycolysis. SIPA1 may alter the main source of ATP production from oxidative phosphorylation to glycolysis by promoting the transcription of EPAS1 (a gene encoding hypoxia-inducible factor-2α (HIF-2α)) and interacting with multiple glycolysis-related genes in breast cancer models. Developing new strategies targeting SIPA1 and blocking aerobic glycolysis may provide promising insight in treating breast cancer patents.

Cancer cell reprogramming supported the existence of CSCs, which are characterized by the permanent processes of differentiation and dedifferentiation. Therefore, there is a need to know more about the molecular regulation of the processes of CSC differentiation/dedifferentiation. The manuscript by Ghuwalewala et al. demonstrates that CD24 is a functional target of miR-146a. miR-146a can modulate CSC properties through CD24-AKT-β-catenin axis. It opens new perspectives in the development of more precise diagnostic tools and personalized medicine approaches to be used in the management of HNSCC patients.

A little part in this issue is an interesting review by Zuo et al., devoted to a special aspect of cell physiology as the presence and functional significance of such underestimated structure as extrachromosomal circular DNA (eccDNA). The authors give a brief discovery history of eccDNA, describe several models of the eccDNA biogenesis and their important role in a variety of normal physiological processes and carcinogenesis. The high stability of extracellular free eccDNAs and their presence in cancer tissues and peripheral blood of cancer patients suggest the potential applying them as a novel type of biomarkers in liquid biopsy for the early detection of diseases, the monitoring of drug treatment response, and cancer survival.

Interestingly, in this Research Topic, there is an article systematically discussed conditional reprogramming (CR) cell culture technology in cell reprogramming research of digestive System diseases. It is based on using irradiated Swiss-3T3-J2 mouse fibroblast cells and the Rho-associated kinase (ROCK) inhibitor Y-27632. This technology may work as a promising model for drug sensitivity tests, gene profile analyses, and even tissue regeneration-associated applications. Never or less, the limitations of CR were also summarized. In particular, the authors’ team shared the experience of establishing the Next Generation Living Biobank (NGLB) based on 2D and 3D culture systems with the potential of combining CR technology (Zhao et al.).

## Conclusion

This Research Topic sought to collect promising translational studies, new mechanisms and molecular signatures, highlighting the importance of understanding the molecular mechanism and the clinical impact of cancer cell reprogramming in carcinogenesis and malignancy progression. Developing new therapeutic strategies and promising approaches which regulate the cancer cell plasticity may provide insights to overcome the therapy resistance and propose successful management of the metastatic disease.

## Author contributions

All authors have made a substantial contribution to this editorial work, and approved the context for publication.
